# A Ticklish Affair: Tickling Impacts Play Features in Bonobos

**DOI:** 10.1002/ajp.70216

**Published:** 2026-10-23

**Authors:** Elisa Demuru, Chiara Andreozzi, Edoardo Collarini, Marta Panunzi, Jean‐Pascal Guéry, Giada Cordoni, Ivan Norscia

**Affiliations:** ^1^ Department of Life Sciences and Systems Biology University of Torino Turin Italy; ^2^ Animal‐Mente (EI) Neydens France; ^3^ Department of Philosophy, Communication and Performing Arts Roma Tre University, Via Ostiense Rome Italy; ^4^ La Vallées des Singes Romagne France

**Keywords:** communication, Gargalesis, intersubjectivity, *Pan paniscus*, Primates, touch

## Abstract

Heavy tickling has been reported only in humans and great apes. Because it requires complex socio‐emotional/cognitive skills, it can be a model for investigating intersubjectivity origins. We examined how tickling influences the temporal, structural, and communicative organization of bonobo social play. We gathered video data from a bonobo group housed at *La Vallée des Singes* (France) over 3 months. By analysing 215 dyadic play sessions (27 dyads), we found that at the temporal level sessions containing tickling lasted longer than those without. Tickling reduced the total duration of pauses, although its effect on pause occurrence depended on object presence. A subset of 45 sessions (11 dyads) underwent detailed frame‐by‐frame analysis. Within this subset, tickling was associated with lower play asymmetry, reduced behavioral diversity, lower evenness, and less repetition, indicating more balanced, focused, yet non‐repetitive interactions. Within the same subset, at the communicative level, tickling did not affect play‐face reciprocity or rapid facial mimicry but was associated with reduced mutual gaze. Overall, tickling modified multiple features of bonobo play by sustaining engagement, shortening pauses, and promoting balanced behavioral participation. The selective reduction in mutual gaze, despite unchanged facial reciprocity and mimicry, suggests that coordination during tickling relies more strongly on tactile exchanges than sustained face‐to‐face visual orientation. By demonstrating how a specific tactile component of social play contributes to the maintenance, coordination, and sensory organization of playful interactions, these findings support tickling as a natural model for investigating the evolutionary foundations of intersubjective multimodal processes predating the human‐Pan split.

## Highlights

- Tickling prolonged bonobo social play sessions and reduced the duration of interruptions, indicating a role in sustaining playful engagement.
- Tickling promoted a more balanced and focused organization of play by reducing behavioral asymmetry, diversity, evenness, repetition and mutual gaze, while leaving play‐face reciprocity and rapid facial mimicry unchanged.
- These findings identify tickling as a valuable natural model for investigating the evolutionary origins of intersubjectivity, and tactile social coordination in hominids.

## Graphical abstract

The image sequences show two mother–offspring tickling interactions in bonobos. (a) A female tickles her son's belly with her hands; he shows a full play face and grasps her finger in a play shelter response. (b) A female tickles her daughter with her mouth; the infant shows a full play face.

## 1 Introduction

At the core of social interaction lies the ability to connect self and others. In psychology, this capacity is conceptualized as intersubjectivity, namely the sense of togetherness that emerges when individuals become aware of and respond to one another's emotions, intentions, and actions, creating a shared space of engagement (Gärdenfors 2007; Zlatev 2008; Rochat 2009). Intersubjectivity is considered fundamental to social relationships, communication, and cooperation, and understanding its development and evolution remains a central challenge in social cognition research. Recent approaches view intersubjectivity not as a single cognitive ability but as a continuum ranging from basic self–other discrimination to more complex forms of joint action and shared intentionality (Heesen et al. 2017; Demuru et al. 2022). From this perspective, higher socio‐cognitive abilities may emerge from simpler processes of emotional attunement, behavioral coordination, and reciprocal engagement that are already observable in non‐human animals (Preston and De Waal 2002; De Waal and Ferrari 2010; Prochazkova and Kret 2017; Norscia 2026). Identifying behavioral contexts that foster such reciprocal adjustment is therefore crucial for understanding the biological roots of intersubjective abilities.

Among the social contexts in which intersubjective processes may emerge, social play is particularly informative because it requires individuals to remain mutually engaged during interactions that are highly variable, challenging, and potentially risky, especially during play fighting. To maintain play, partners must continuously monitor and adjust to each other's behavior, regulate the intensity of their actions, and preserve a shared understanding of the playful nature of the interaction, thereby reducing the risk of escalation into aggression (Palagi 2006; Cordoni and Palagi 2011; Pellis et al. 2024). This risk arises because many play‐fighting behaviors are derived from the aggressive repertoire (Cordoni et al. 2023; Pellis et al. 2024; Cordoni and Norscia 2025). In primates, play‐specific facial expressions, such as the play face and full play face (Pellis and Pellis 1996), help reduce this ambiguity by conveying the sender's emotional and motivational state and signalling playful rather than aggressive intent (Waller and Dunbar 2005; Palagi 2008). Their expression, perception, and matching facilitate behavioral coordination, and studies on Rapid Facial Mimicry (RFM)—the replication of a partner's facial expression within 1 s (Palagi et al. 2020)—have linked it to increased synchrony and longer play sessions (Mancini et al. 2013; Palagi et al. 2016). Because play relies on active participation and reciprocal responsiveness, it has long been considered a valuable context for investigating the behavioral foundations of coordinated joint actions (Heesen et al. 2021). This is particularly evident in dyadic play, where interaction depends entirely on the continuous behavioral adjustments of both partners, making it an ideal natural context for investigating coordination, mutual engagement, and the behavioral foundations of intersubjectivity (Demuru et al. 2022). Within the broad repertoire of social play behaviors, tickling represents a distinctive pattern. Here, we refer specifically to *gargalesis*, a vigorous form of tactile stimulation embedded in social and playful interactions, as opposed to the lighter *knismesis*, which is more commonly associated with the detection of external stimuli on the body surface (Harris 2012). Gargalesis involves repeated stimulation of specific body areas and typically elicits defensive responses accompanied by specific laughter‐like vocalizations, body movements, withdrawal attempts, and/or renewed engagement (Hall and Allin 1897; Harris 1999; Provine 2001, 2004; Szameitat et al. 2009). Because the present study focuses on social play, the term *tickling* hereafter refers exclusively to gargalesis. Another distinctive feature of tickling is that it is remarkably difficult to self‐induce, as self‐generated tactile stimulation does not normally evoke the characteristic behavioral and emotional responses associated with it (Leuba 1941; Weiskrantz et al. 1971; Ishiyama et al. 2019). This asymmetry is thought to result from predictive sensorimotor mechanisms that attenuate the sensory consequences of one's own actions while preserving sensitivity to externally generated stimulation (Blakemore et al. 1999, 2000; Harris 1999; Wolpert and Ghahramani 2000; Kilteni 2025). Notably, the efficiency of this mechanism varies with schizotypy and may be altered in psychotic patients, some of whom can tickle themselves, suggesting a reduced distinction between self‐generated and externally generated sensory events (Whitford et al. 2017).

Thus, from an intersubjective perspective, tickling is particularly relevant because it highlights one of the most fundamental dimensions of social cognition: the distinction between self and other. This aspect becomes clear when the factors that modulate tickling are examined. Beyond its relevance as an indicator of the capacity for self–other distinction in humans, tickling also represents a highly social form of interaction whose occurrence, development, and expression are remarkably similar in hominids (humans and great apes), thereby offering a valuable opportunity to investigate the evolutionary origins of such capacity. The available evidence, in a comparative perspective, suggests that tickling shares a number of common features across hominids, as it has only been reported in humans and great apes. Such limited presence in primates suggests that such behavior requires complex socio‐cognitive skills. Tickling laughter response is conserved across hominids (orangutans, gorillas, bonobos, chimpanzees, humans; De Gregorio et al. 2026). In humans, chimpanzees, and bonobos (hominins), tickling is strongly embedded within social interactions, occurs predominantly between highly affiliated partners, and is particularly common during caregiver–offspring interactions (Provine 1997; Bard et al. 2014; Leavens and Bard 2016; Demuru, Montello, et al. 2025). Across all three species, individuals initially experience tickling as recipients and only later become able to anticipate, solicit, and direct tickling towards others, revealing a similar developmental trajectory from passive reception to active participation in the interaction (Provine 1997; Bard et al. 2014; Leavens and Bard 2016; Ishijima and Negayama 2017; Negayama et al. 2021; Demuru, Montello, et al. 2025). Furthermore, tickling is organized around repeated cycles of tactile stimulation and behavioral response and is shaped by the social characteristics of the interacting partners rather than by the physical stimulation alone (Robinson 1907; Provine 1997; Ikeda and Itakura 2013). Together, these similarities suggest that tickling represents an evolutionarily conserved form of social interaction at least in the hominin tribe within the hominid family, characterized by strong affiliative value, developmental significance, and reciprocal engagement between partners (Provine 1997; Bard et al. 2014; Leavens and Bard 2016; Demuru, Montello, et al. 2025). However, despite the growing literature on the development and social functions of tickling, almost nothing is known about how tickling influences the organization of ongoing play interactions.

Social play can be viewed as a form of joint action in which participants continuously adjust their behavior to maintain a shared interaction (Heesen et al. 2017, 2021). Thus, tickling—by requiring partner‐specific responsiveness, reciprocal engagement, and repeated action/reaction exchanges—may actively shape the temporal organization, behavioral structure, and communicative exchanges of playful interactions. Temporal organization—in particular, the way interactions are maintained, interrupted, and resumed—reflects the ability of partners to remain engaged in a shared activity and to restore playful engagement following interruptions (Heesen et al. 2017, 2021). Beyond temporal persistence, social play can differ in how behavioral exchanges are organized. This internal organization can be described through complementary indices that capture behavioral symmetry, repetition, diversity, and evenness of the play patterns within sessions (Cordoni and Palagi 2016; Cordoni et al. 2018; Cordoni and Norscia 2025). A balanced exchange of offensive and defensive behaviors reflects reciprocal participation, whereas behavioral repetition, diversity and evenness describe complementary aspects of behavioral organization, including the tendency to repeatedly perform the same actions, the richness of the behavioral repertoire, and how evenly different behaviors are distributed throughout a session (Cordoni et al. 2022, 2023). Together, these complementary indices provide a multidimensional description of play organization and allow assessment of whether tickling influences not only how long play lasts but also how behavioral exchanges are organized (Cordoni and Norscia 2025).

Finally, communicative exchanges provide an additional mechanism through which partners coordinate their behavior during social play. Visual signals, including facial expressions and gaze, help players monitor one another's actions, communicate playful intentions, and continuously adjust their behavior during the interaction (non‐human primates; Waller and Dunbar 2005; Mancini et al. 2013; Palagi et al. 2016). Play‐face reciprocity and RFM reflect reciprocal facial communication, whereas mutual gaze represents one of the clearest behavioral indicators of reciprocal visual orientation and partner monitoring (Emery 2000; Amici et al. 2025). Although tickling primarily involves tactile interactions, successful tickling episodes occur within broader multimodal exchanges involving gaze, facial expressions, and caregiver responsiveness (humans; Ikeda and Itakura 2013; Negayama et al. 2021).

Bonobos provide a suitable model for investigating how tickling shapes ongoing play interactions and the possible evolutionary implications of this role because they are the closest living relatives of *Homo sapiens* (together with chimpanzees) and exhibit advanced socio‐cognitive abilities, and a particularly rich repertoire of affiliative and playful behaviors (Kano 1992; Palagi 2006; Demuru et al. 2015; Gruber and Clay 2016). However, despite the growing literature on the development and social functions of tickling, nothing is known about how tickling influences ongoing play interactions. Overall, the elements outlined above suggest that its potential effects can be examined across temporal organization, behavioral structure, and communicative exchanges. On this basis, we formulated a set of predictions at two complementary levels: a macro level, focusing on session features such as duration and pauses, and a micro level, focusing on behavioral and communicative dynamics unfolding within play sessions.

### 1.1 Prediction 1—Effects of Tickling on Play Duration

Because tickling relies on the active involvement and reciprocal responsiveness of both partners, and is typically embedded within highly affiliative social relationships (van de Rijt‐Plooij and Plooij 1987; Bard et al. 2014; Ishijima and Negayama 2017; Demuru, Montello, et al. 2025), we predicted that play sessions containing tickling would last longer than sessions in which tickling was absent, reflecting the higher social engagement associated with this behavior.

### 1.2 Prediction 2—Effect of Tickling on Play Interruptions

Because tickling relies on successive episodes of engagement and re‐engagement interspersed with brief regulatory intervals that allow players to adjust their behavior (Leuba 1941; van Boekholt et al. 2024; Kilteni 2025), we predicted that interruption dynamics (i.e., presence, duration and occurrence of pauses) would be more pronounced in sessions containing tickling than in sessions without it.

### 1.3 Prediction 3—Effects of Tickling on Play‐Session Structure

As said above, tickling has been proposed to promote the active involvement and reciprocal responsiveness of both play partners (van de Rijt‐Plooij and Plooij 1987; Bard et al. 2014; Ishijima and Negayama 2017; Demuru, Montello, et al. 2025). Greater mutual involvement and reciprocity are generally associated with longer play sessions, which in turn tend to be more symmetrical in structure (Palagi and Cordoni 2012; Cordoni et al. 2018). We therefore predicted that, by enhancing partner involvement and reciprocal responsiveness, sessions with tickling—compared to sessions without it—would be associated with lower levels of play asymmetry (*Prediction 3a*). Because tickling itself is a highly repetitive behavior that relies on the reiterated alternation between tactile stimulation and specific behavioral responses from the recipient (Leuba 1941; Selden 2004; Bard et al. 2014; Demuru, Montello, et al. 2025; Kilteni 2025), we predicted that sessions containing tickling—compared to sessions without it—would be characterized by greater behavioral repetition (*Prediction 3b*), and lower behavioral variability (*Prediction 3c*). No a priori prediction could be formulated for behavioral evenness.

### 1.4 Prediction 3—Effects of Tickling on Facial Communication

Although tickling is primarily mediated through tactile interactions, successful tickling episodes unfold within a broader multimodal exchange involving facial expressions, gaze, and continuous behavioral adjustment between partners (Ikeda and Itakura 2013; Negayama et al. 2021). Because visual communicative mechanisms such as play‐face reciprocity, RFM, and mutual gaze facilitate reciprocal engagement and the accurate interpretation of playful intentions (Waller and Dunbar 2005; Mancini et al. 2013; Demuru et al. 2015; Palagi et al. 2016; Palagi et al. 2019; Cordoni et al. 2024; Cordoni et al. 2025), and because tickling is embedded within broader communicative exchanges involving facial and visual cues (Ikeda and Itakura 2013; Negayama et al. 2021), we predicted that sessions containing tickling would exhibit higher levels of social coordination than sessions without tickling. Specifically, if tickling increases the need for partner monitoring, behavioral adjustment, and the maintenance of a shared playful interaction, visual communicative mechanisms supporting social coordination should occur more frequently. Accordingly, we expected higher rates of play‐face reciprocity (*Prediction 3a*), RFM (*Prediction 3b*), and mutual gaze (*Prediction 3c*).

## 2 Materials and Methods

### 2.1 Ethics Statement

This investigation was entirely observational and non‐manipulative, therefore, approval from the Bio‐Ethical Committee of the authors' institutions was not required. This investigation is compliant with the American Society of Primatologists Principles for the Ethical Treatment of Non‐Human Primates.

### 2.2 Study Site and Subjects

The study was conducted at La Vallée des Singes (Romagne, France) on a captive group of 20 bonobos (*Pan paniscus*) ranging in age from 1 to 55 years (mean ± SD = 16.10 ± 14.11 years). Following the age classification adopted by Demuru, Montello, et al. (2025), individuals were assigned to three age classes: infants (0–5 years), juveniles (after the weaning period; 6–10 years), and adults (≥ 11 years). The group included 13 adults (8 females and 5 males), 2 juveniles (1 female and 1 male), and 5 infants (4 females and 1 male). Maternal kinship relationships were known for all individuals (Table 1). Bonobos were housed in a combined indoor–outdoor enclosure consisting of an approximately 500 m^2^ indoor facility and a naturalistic outdoor island of approximately 1 ha. Although the enclosure was designed to allow group subdivision, bonobos were managed as a stable social group. During the study period, paper sheets and clothing items were regularly provided as environmental enrichment.

**Table 1:** Study group composition and individual features.

Name	Sex	Date of birth	Age class	Maternal Kinship
Daniela	F	17/06/1968	Adult	Mother of Diwani and David
Diwani	M	11/08/1996	Adult	Son of Daniela and brother of David
David	M	27/07/2001	Adult	Son of Daniela and brother of Diwani
Khaya	F	19/10/2001	Adult	Mother of Khalessi and Kemba
Lingala	F	17/07/2003	Adult	Mother of Kipepeo
Ukela	F	19/12/1985	Adult	Mother of Moko, Kymia, and Lukombé
Kelele	M	22/07/2004	Adult	—
Lucy	F	01/12/2003	Adult	Mother of Bembé
Moko	M	04/08/2012	Adult	Son of Ukela, brother of Kymia and Lukombé
Khalessi	F	12/12/2012	Adult	Daughter of Khaya and sister of Kemba
Ulindi	F	10/10/1993	Adult	Mother of Loto and Lokoro
Loto	M	02/09/2009	Adult	Son of Ulindi and brother of Lokoro
Yahimba	F	07/08/2009	Adult	Mother of Ybambé
Lokoro	M	22/05/2015	Juvenile	Son of Ulindi and brother of Loto
Kymia				
	F	12/04/2017	Juvenile	Daughter of Ukela, sister of Moko and Lukombé
Lukombé	M	03/04/2022	Infant	Son of Ukela, brother of Moko and Kymia
Bembé	F	26/05/2022	Infant	Daughter of Lucy
Ybambé	F	07/12/2022	Infant	Daughter of Yahimba
Kipepeo	F	22/12/2022	Infant	Daughter of Lingala
Kemba	F	24/06/2022	Infant	Daughter of Khaya and sister of Khalessi

### 2.3 Data Collection and Operational Definitions

Behavioral data were collected between October and December 2023 from approximately 10:00 a.m. to 5:30 p.m., 5 days per week. Due to the low temperatures characterizing the study period, bonobos spent most of their time indoors, although they had regular access to the outdoor space. Observations were conducted through large observation windows located on the upper floor of the facility and, when necessary, from keeper‐only areas on the lower level. Prior to data collection, the data collector (C.A.) underwent a training period under the supervision of E.D. for reliable coding and reached a substantial to almost perfect inter‐observer agreement (Cohen's *κ* mean = 0.856; range = 0.720–1.000; confusion matrix reported in Figure S1). Playful interactions were video‐recorded using Full HD video cameras (Panasonic HC‐V180 Handy Cam, 90× zoom, 50 fps) and subsequently analysed frame‐by‐frame using Avidemux 2.8.

Using the all‐occurrences animal sampling method (Altmann 1974) we collected a full dataset of 215 play sessions—including 107 sessions with at least one interruption (hereafter “pauses”)—, involving 27 dyads. Only dyadic play sessions, recorded from beginning to end and in which both participants remained visible throughout the interaction, were retained. Recordings started with the first play pattern and ended with the last (see below for play definition), continuing for at least two additional minutes whenever possible to detect potential re‐engagement between the same players. To investigate temporal dynamic of play sessions, we extracted from videos the following information: identity of the participants (hereafter “dyad identity”), individual features (sex, age class), occurrence of tickling, occurrence of play with object (hereafter “object play”), play session duration (*s*), occurrence of pauses, total duration of pauses and total number of pauses. Subsequently, from the full dataset, we derived a second subset of 45 play sessions involving 11 dyads to investigate play structure, facial communication, and visual coordination. We retained sessions in which both participants remained continuously visible and facial visibility was possible throughout the session. For this purpose, we extracted from videos the following information: play behavioral patterns, facial expressions, visual communicative patterns (Table 2). To investigate communicative characteristics, we also quantified simultaneous face visibility time for each dyadic play session, defined as the amount of time during which both players' faces were simultaneously visible. This variable was estimated using scan sampling at 30 *s* intervals (Altmann 1974), with a minimum value of 1 *s* assigned to each session, and was used to account for differences in the opportunity to observe facial and visual communicative behaviors.

**Table 2:** Operational definitions of behavioral patterns, facial expressions, and visual communication variables coded during dyadic play sessions.

Behavior	Code	Definition	Category
**Play behavioral patterns**			
Arm Wrestling	AWREST	Two individuals alternately slap each other's raised arms while standing bipedally.	O
Attempt Play Bite	APBIT	An individual attempts to bite the play partner but fails to make contact.	O
Attempt Play Grab	APGR	An individual attempts to grasp the play partner but fails to establish contact.	O
Play Bite	PBIT	An individual gently bites the play partner without causing injury.	O
Play Brusque Rush	PBR	An individual jumps onto the partner using all four limbs during play.	O
Play Crouching	PCR	An individual crouches in front of the play partner in a self‐handicapping posture.	D
Play Climb	PCLIMB	An individual climbs onto or stands on the partner's body without using it as support for jumping.	N
Acrobatic Play	ACP	One or more individuals perform acrobatic movements by swinging or jumping on environmental structures.	N
Airplane	AIR	An adult supports an infant above the body using the hands or feet while lying on the ground.	N
Swinging Over Partner	DONS	An individual swings above the partner, often while inviting the partner to interact.	N
Head Right–Left	HEADRL	An individual moves the head horizontally from side to side, sometimes during tickling or playful biting.	N
Play Bottom Up	PBU	An individual adopts a play posture with extended hind limbs and flexed forelimbs, exposing the flanks to the partner.	N
Arm Wrestling	AWREST	Two individuals alternately slap each other's raised arms while standing bipedally.	O
Attempt Play Bite	APBIT	An individual attempts to bite the play partner but fails to make contact.	O
Play Eye Cover	PEYEC	An individual covers the partner's eyes using the hand, foot, or an object.	N
Play Finger in Mouth	PLAYFM	An individual inserts one or more fingers, or the hand, into the partner's mouth.	N
Play Grab	PGR	An individual grasps the partner using the hands or feet while maintaining contact.	O
Play Kick	PKICK	An individual strikes the partner using one or both hind limbs.	O
Play Push	PPS	An individual pushes the partner using the hands or feet.	O
Play Pull	PPU	An individual pulls the partner towards itself using the hands or feet.	O
Play Retrieve	PRE	An individual restrains the partner to prevent it from moving away.	O
Play Slap	PSL	An individual slaps any part of the partner's body with the hand.	O
Play Shelter	PLAYSH	An individual protects itself from playful attacks using the hands, feet, or an object.	D
Play Wriggle	PLAYWR	An individual twists and wriggles to escape from the partner's grasp.	D
Play Roll	PRO	An individual rolls its body from side to side while lying on its back.	N
Play Twirl	PTW	An individual rotates around its own body axis while standing bipedally.	N
Rough‐and‐Tumble	R&T	Two individuals engage in dynamic wrestling that includes combinations of chasing, rolling, grabbing, slapping, and playful biting.	N
Tickle	TK	An individual repeatedly grasps, probes, or presses the partner's ticklish body regions (i.e., neck, belly, feet, armpits) using the hands, feet, or mouth, eliciting a characteristic ticklish response.	N
Watching Back	WB	An individual turns around to visually monitor the play partner.	N
**Facial expressions**			
Play Face	PF	An individual displays a relaxed open‐mouth expression with only the lower teeth exposed.	
Arm Wrestling	AWREST	Two individuals alternately slap each other's raised arms while standing bipedally.	
Attempt Play Bite	APBIT	An individual attempts to bite the play partner but fails to make contact.	
Full Play Face	FPF	An individual displays a relaxed open‐mouth expression with both upper and lower teeth exposed.	
**Visual communication**			
Mutual Gaze	MG	Two individuals simultaneously orient their face and gaze towards each other, establishing direct visual contact during the play interaction.	

A session was defined as a playful interaction whose behavioral repertoire included at least one play‐specific behavior or play signal (Table 2). The play session was considered to begin when one individual directed a playful pattern towards a social partner and the partner responded with a playful behavioral pattern, thereby establishing a reciprocal playful exchange. A session ended with the last play pattern performed before the interaction ceased without resumption, one participant disengaged and moved away, or the composition of the interaction changed following the intervention of a third individual who interrupted the interaction, replaced one of the players, or joined the ongoing play session. Recordings started with the first play pattern and ended with the last, continuing for at least two additional minutes whenever possible to detect potential re‐engagement between the same players.

When the play sessions were interrupted and subsequently resumed by the same partners, interruption durations were measured to distinguish temporary pauses from the termination of a play session. Using the third quartile (75th percentile) of the interruption‐duration distribution as a threshold (Q3 = 47.18 s), interruptions lasting 48 *s* or less were classified as pauses within the same play session, whereas longer interruptions were treated as session breaks.

In bonobos, tickling was defined as a repeated grasping, probing, or pressing action performed with the hands, feet, or mouth and directed towards body regions commonly associated with ticklish responses, including the neck, belly, feet, and armpits (Demuru, Montello, et al. 2025; Figure 1; Video S1). A tickling occurrence began when contact with one of these body regions was established and ended when contact ceased. Consecutive tickling occurrence separated by less than 5 *s* were considered part of the same tickling sequence, following Demuru, Montello, et al. (2025). For each tickling event, actor identity, recipient identity, and duration were recorded.

**Figure 1:**
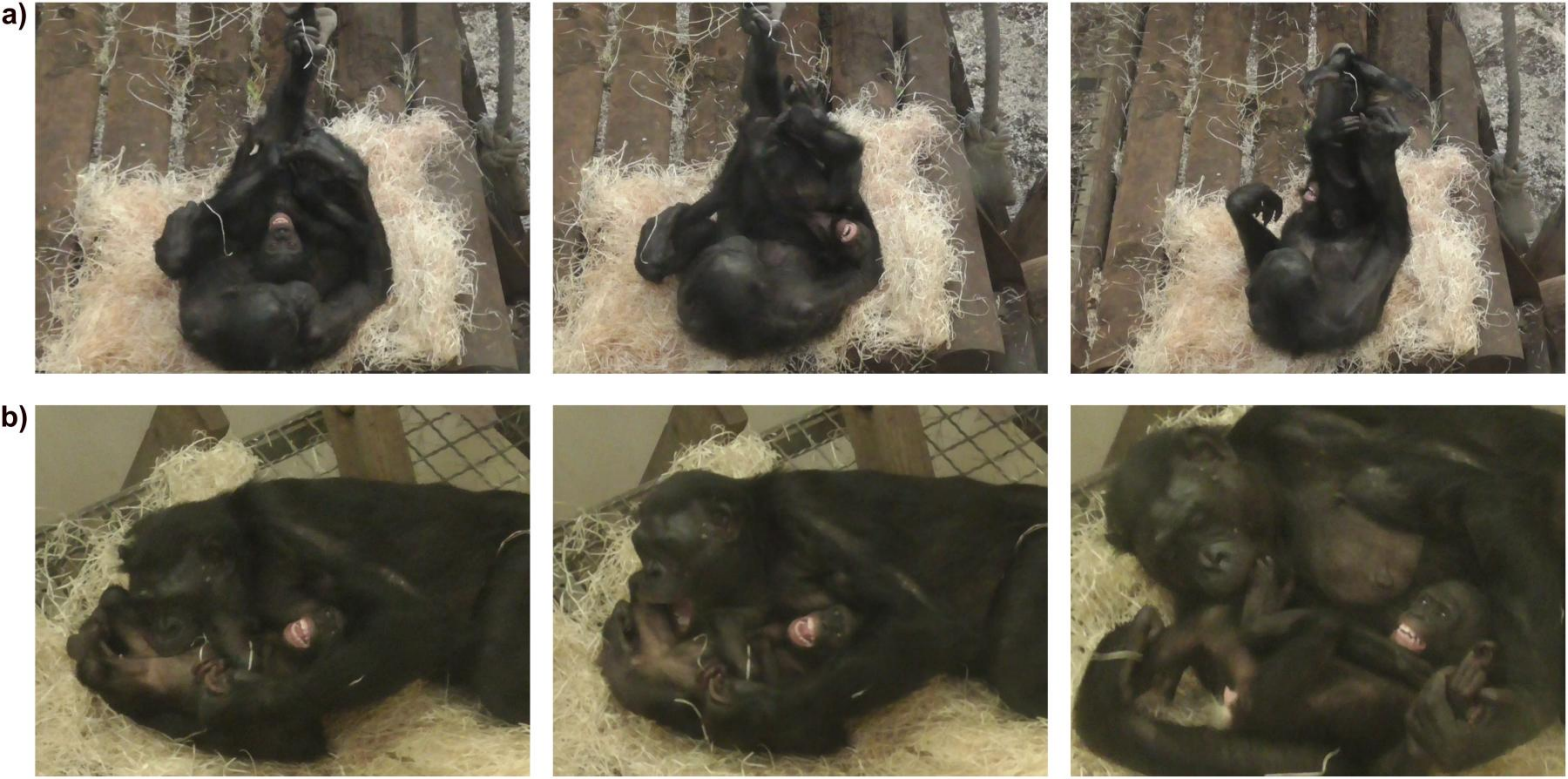
The image sequences show, from left to right in each row, two mother–offspring tickling interactions in bonobos. (a) An adult female tickles her infant son with her hands on the belly; during the sequence, a full play face is visible, and the infant grasps the mother's finger in a play shelter response, apparently attempting to interrupt or prevent the tickling stimulation. (b) An adult female tickles her infant daughter using her mouth on the belly, inner thigh, and foot; the infant responds with a full play face.

During video analyses, we coded play facial expressions, namely play face and full play face (Table 2). Play face was defined as open‐mouth expression with the lower teeth exposed (van Hooff and Preuschoft 2003), whereas full play face was defined as a relaxed open‐mouth expression with both upper and lower teeth exposed (Davila‐Ross and Dezecache 2021). RFM was defined as the production of a congruent play face or full play face within 1 s of perceiving a partner's play face expression, following previously adopted criteria in studies of primate play communication (Davila‐Ross et al. 2009; Palagi et al. 2016; Davila‐Ross and Palagi 2022). Mutual gaze was operationally defined as a proxy for a face‐to‐face orientation, in which the two individuals were facing each other frontally (i.e., not at an oblique angle) and the eyes of both were visible to the observer. Cases (*n* = 17) in which there was no such visibility to assess mutual gaze were excluded from the analysis.

#### 2.3.1 Play Face Reciprocity Index (PFRI)

To quantify the reciprocity of facial expressions exchanged during play sessions, we calculated a PFRI based on the directional distribution of play faces exchanged between players. The index was designed to distinguish between the absence of facial signalling, unilateral facial signalling, and reciprocal facial exchange within a session. Sessions in which neither player produced a play face were assigned a value of 0. When play faces were produced by only one player (unilateral facial signalling), the index generated values strictly below 0.5, reflecting unilateral facial signalling, according to the following formula:

$$\mathrm{PFRI}=0.5*[\frac{\left(\mathrm{PF}\left(A\to B\right)+\mathrm{PF}\;\left(B\to A\right)\right)\;}{\mathrm{PF}\left(A\to B\right)+\mathrm{PF}\;\left(B\to A\right)+1}]$$

where PF(A → B) and PF(B → A) represent the number of play faces emitted by player A towards player B and by player B towards player A, respectively. In this unilateral context, the numerator represents the total unreciprocated signaling effort, while the denominator represents the total volume of facial signaling plus a smoothing factor (+1). The inclusion of a smoothing factor (+1) in the denominator of the unilateral formula ensures that the index accounts for the total volume of signaling, allowing the value to asymptotically approach 0.5 as the number of unreciprocated play faces increases; this mathematically reflects a higher potential communicative investment by the single active player despite the lack of partner response.

When both players displayed at least one play face (bilateral facial signalling), reciprocity was calculated as:

$$\mathrm{PFRI}=0.5+0.5\;*\;[\frac{2\;*\;\min\left(\mathrm{PF}\left(A\to B\right),\;\mathrm{PF}\;\left(B\to A\right)\right)\;}{\mathrm{PF}\left(A\to B\right)+\mathrm{PF}\;\left(B\to A\right)\;}]$$

The term “min” in the the numerator min(PF(A → B), PF(B → A)) corresponds to the smaller of the two values and quantifies the reciprocated component of facial signalling, whereas the denominator PF(A → B) + PF(B → A) represents the total amount of facial signalling produced during the session (the 2x factor ensures the index to assume 1 as maximum value). The baseline value of 0.5 is added to create a strict mathematical threshold that categorically separates unilateral interactions (capped below 0.5) from bilateral ones (ranging from strictly above 0.5 to 1). Furthermore, the smoothing factor (+1) is omitted in this bilateral formula because both players displayed at least one play face (PF > 0), ensuring that the ratio perfectly reflects the true symmetry of the exchange without any structural underestimation or mathematical dampening.

The index ranges from 0 to 1. A value of 0 indicates the total absence of facial signalling, values between 0 (exclusive) and 0.5 indicate unilateral facial signalling, whereas values greater than 0.5 indicate bilateral facial signalling, with increasing values corresponding to increasing reciprocation level. Finally, a value of 1 indicates perfect reciprocity, whereby both players display the same number of play faces.

#### 2.3.2 Play Structure Indices

To investigate whether tickling was associated with changes in the internal organization of play sessions, based on Cordoni and Palagi (2016; Cordoni et al. 2018) we calculated the Play Asymmetry Index (pAI) and based on Collarini et al. (2022) we calculated three further indices describing different structural properties of play interactions: Repetition Index (RI), Shannon Diversity Index (H′), and Pielou Evenness Index (J).

pAI measures the degree of asymmetry in social play sessions by comparing the balance of offensive and defensive patterns exchanged between partners and ranges from −1 to +1, with 0 indicating complete symmetry and extreme values indicating complete asymmetry in favor of one player. In this case defensive patterns were those aimed at protecting one's own body (e.g., sheltering) and thus self‐directed, whereas offensive patterns were those aimed at hitting or otherwise acting upon the playmate (e.g., slap). It was calculated as:

$$p\mathrm{AI}=\frac{\left({\mathrm{offensive}}_{A\to B}+{\mathrm{defensive}}_{B\to A}\right)-\left({\mathrm{offensive}}_{B\to A}+{\mathrm{defensive}}_{A\to B}\right)}{\left({\mathrm{offensive}}_{A\to B}+{\mathrm{defensive}}_{B\to A}\right)+\left({\mathrm{offensive}}_{B\to A}+{\mathrm{defensive}}_{A\to B}\right)+{\mathrm{neutral}}_{A+B}\;}$$

The RI quantified the tendency of players to repeatedly perform the same behavioral patterns within a session. It was calculated as the proportion of repeated behavioral pattern types relative to the total number of behavioral patterns performed during the session, normalized by session duration. Higher RI values indicate a greater tendency to reiterate previously performed behaviors, reflecting lower behavioral flexibility.

To characterize behavioral diversity, each play session was treated as an ecosystem in which behavioral patterns included in the session represented different “species” and individual behavioral events represented their occurrences. H′ quantified the diversity of behavioral richness (number of behavioral pattern types) and their relative abundance within a session. It was calculated as:

$$H^{\prime }=-\Sigma \left[\left(\frac{n_{i}}{N}\right)*\left(\frac{\ln n_{i}}{N}\right)\right]$$

where $n_{i}$ is the frequency of pattern type *i* and *N* is the total number of patterns in the session. Higher H′ values indicate greater behavioral diversity. Pielou's evenness index (J), derived from Shannon's index, measures how evenly behavioral patterns are distributed among the different pattern types. It was calculated as:

$$J=H\prime {/H\prime }_{\max}$$

where *H′* is the observed Shannon Diversity Index and *H*'max is the total number of different behavioral pattern types. J ranges from 0 to 1, with higher values indicating a more even distribution of patterns.

### 2.4 Statistical Elaboration

All statistical analyses were conducted in R (version 4.5.1; R Core Team 2025). Generalised Linear Mixed Models (GLMMs) were fitted using the *glmmTMB()* function from the *glmmTMB* package (Brooks et al. 2017). To investigate the temporal characteristics of play sessions, four GLMMs were fitted to analyse play duration (on *n* = 215 sessions; GLMM_1a_), and pauses (*n* = 107), including pause occurrence (GLMM_1b_), total pause duration (GLMM_1c_), and pause number (GLMM_1d_). To investigate the structural and communicative characteristics of play, seven additional GLMMs were fitted (on *n* = 45 play sessions). Four models assessed play structure, namely the pAI (GLMM_2a_), RI (GLMM_2b_), H′ (GLMM_2c_), and J (GLMM_2d_). Three further models assessed facial communication during play, namely the PFRI (GLMM_2e_), RFM (GLMM_2f_), and Mutual Gaze (MG; GLMM_2g_).

#### 2.4.1 GLMMs on Tickling, Play Duration, and Pauses

The response variables included play‐session duration (GLMM_1a_), pause occurrence (GLMM_1b_), total pause duration (GLMM_1c_), and pause number (GLMM_1d_), respectively. The fixed effects included in all full models were object play (presence/absence), tickling (presence/absence), their interaction, sex combination (FF; mixed‐sex FM/MF; MM), age‐class combination (same age class/different age class), and kinship category (2: mother–infant, 1: siblings, 0: non‐kin). Since the dynamics of play sessions can be influenced by object presence (Shimada 2006) as well as by the sex and age of the individuals involved (Cordoni et al. 2018), we included object play (presence/absence), dyadic sex combination, and age‐class combination as control predictors in the corresponding reduced models. Dyad identity was included as a random effect to account for the non‐independence of observations and repeated measures within dyads. In the models examining pause occurrence, total pause duration, and pause number (GLMM_1b–d_), effective play duration excluding pauses (play duration minus total pause duration) was included as a log‐transformed offset to control for differences in temporal opportunity among play sessions (Table 3).

**Table 3:** Summary of the GLMMs used to test the effect of tickling on play temporal structure, behavioral organization, and facial/visual communication.

Model	Target variable	Fixed variables	Random variable	Offset variable
GLMM_1a_	Play session duration	Object play, tickling, sex combination, age class combination, kinship	Dyad identity	a
GLMM_1b_	Pause occurrence	Object play, tickling, sex combination, age class combination, kinship	Dyad identity	log(Effective play duration)
GLMM_1c_	Total pause duration	Object play, tickling, sex combination, age class combination, kinship	Dyad identity	log(Effective play duration)
GLMM_1d_	Total pause number	Object play, tickling, sex combination, age class combination, kinship	Dyad identity	log(Effective play duration)
GLMM_2a_	Play Asymmetry Index	Tickling	Dyad identity	log(Play session duration)
GLMM_2b_	Repetition Index	Tickling	Dyad identity	log(Play session duration)
GLMM_2c_	Shannon Diversity Index	Tickling	Dyad identity	log(Play session duration)
GLMM_2d_	Pielou Evenness Index	Tickling	Dyad identity	log(Play session duration)
GLMM_2e_	Play Face Reciprocity Index	Tickling	Dyad identity	log(Simultaneous face visibility time)
GLMM_2f_	Rapid Facial Mimicry	Tickling	Dyad identity	log(Simultaneous face visibility time)
GLMM_2g_	Mutual Gaze	Tickling	Dyad identity	log(Simultaneous face visibility time)

a No offset included.

The distributional assumptions of each response variable were evaluated by comparing candidate distributions using goodness‐of‐fit statistics and Akaike Information Criterion (AIC), followed by simulation‐based residual diagnostics (DHARMa; Hartig 2022) and assessment of model convergence. Based on overall model fit, residual diagnostics, and convergence, play duration (GLMM_1a_) was analysed using a Lognormal distribution, pause occurrence (GLMM_1b_) using a Binomial distribution with a logit link, total pause duration (GLMM_1c_) using a Gamma distribution with a log link, and pause number (GLMM_1d_) using a Negative Binomial distribution (*nbinom2* parameterisation) with a log link. Although a Lognormal distribution provided the best distributional fit for total pause duration (GLMM_1c_), the Gamma model was retained because it showed better convergence and residual diagnostics. Detailed results of the distributional screening are provided in Table S1 and Figure S2.

#### 2.4.2 GLMM_S_—Tickling, Play Session Structure, and Facial Communication

The response variables included pAI (GLMM_2a_), RI (GLMM_2b_), H′ (GLMM_2c_), J (GLMM_2d_), PFRI (GLMM_2e_), RFM (GLMM_2f_), and Mutual Gaze (MG; GLMM_2g_), respectively. The fixed effect included in all models was tickling condition (presence/absence). Dyad identity was included as a random effect to account for the non‐independence of observations and repeated measures within dyads. For GLMM_2a–d_, log‐transformed play session duration was included as an offset term to control for differences in observation time across play sessions. For GLMM_2e–g_, log‐transformed simultaneous face visibility time was included as an offset term to account for differences in opportunities to observe facial interactions across play sessions (Table 3).

The distributional assumptions of each response variable were evaluated by comparing candidate distributions using goodness‐of‐fit statistics and AIC, followed by simulation‐based residual diagnostics (DHARMa; Hartig 2022) and assessment of model convergence. Based on overall model fit, residual diagnostics, and computational stability, pAI (GLMM2a), RI (GLMM2b), H′ (GLMM2c), and PFRI (GLMM2e) were analysed using Gaussian distributions with identity links; J (GLMM2d) was analysed using a Beta distribution with a logit link; RFM (GLMM2f) was analysed using a Poisson distribution with a log link; and Mutual Gaze (GLMM2g) was analysed using a Negative Binomial distribution (*nbinom2* parameterisation) with a log link. Although Beta distributions provided the best initial fit for RI and PFRI, Gaussian models were retained because they showed better convergence and residual diagnostics. Likewise, despite the initial evaluation of a Negative Binomial distribution for RFM, the final Poisson model provided greater numerical stability and satisfactory residual behavior. Detailed results of the distributional screening are provided in Table S1 and Figures S3 and S4.

For all models across both analytical blocks, multicollinearity among predictors was assessed using the Variance Inflation Factor (VIF). Collinearity diagnostics were obtained using the *performance* package (Lüdecke et al. 2021). The overall significance of the fixed effects was evaluated by comparing each full model with a corresponding null model using likelihood‐ratio tests (LRTs; *anova()*, test = “*Chisq*”; Forstmeier and Schielzeth 2011). Effect sizes were calculated using the *effectsize* package (Ben‐Shachar et al. 2020; Lüdecke et al. 2020), which allows for the derivation of standardized metrics specifically tailored to the structure of generalized linear mixed models. The contribution of individual predictors was subsequently examined using single‐term deletion procedures. Estimated marginal means were calculated using *emmeans* package (Lenth 2025), and model parameters were extracted using *parameters* package (Lüdecke et al. 2020) and visualised using *ggplot2* (Wickham 2016). Statistical significance was set at *p* < 0.05 (Table 4).

**Table 4:** GLMMs on the influence of object play, tickling, sex combination, age class combination and kinship on play session duration (GLMM_1a_), pause occurrence (GLMM_1b_), total pause duration (GLMM_1c_), and total pause number (GLMM_1d_); GLMMs on the effect of tickling on play session structure indices (Play Asymmetry Index, Shannon Diversity Index, Repetition Index, and Pielou Evenness Index; GLMM_2a–d_) and facial communication (Play Face Reciprocity Index, Rapid Facial Mimicry, and mutual gaze; GLMM_2e–g_).

Predictors	Estimates	SE	CI95	*z*	*p*
**GLMM**_**1a**_—**Play session duration:** ***n*** = **215; full vs null: *χ*** ^**2**^ = **30.15; *df*** = **4;** ***p*** < **0.001**					
(Intercept)	5.03	0.25	a	a	a
Object play [Y]^b^	0.06	0.15	−0.23, 0.35	0.41	0.682
Tickling [Y]^b^	0.57	0.10	0.37, 0.77	5.59	**< 0.001**
Sex combination [MIX]^b^	−0.03	0.18	−0.38, 0.32	−0.16	0.874
Sex combination [MM]^b^	−0.08	0.24	−0.55, 0.40	−0.31	0.757
Age‐class combination [Different]^b^	−0.32	0.18	−0.67, 0.04	−1.74	0.082
Kinship [1]^b^	0.19	0.17	−0.15, 0.53	1.09	0.274
Kinship [2]^b^	0.14	0.19	−0.23, 0.50	0.72	0.473
Object play × Tickling	−0.40	0.22	−0.82, 0.03	−1.82	0.069
**GLMM**_**1b**_—**Pause occurrence:** ***n*** = **215; full vs. null: *χ* ^2^** = **10.69; *df*** = **4;** ***p*** = **0.030**					
(Intercept)	−4.47	0.93	a	a	a
Object play [Y]^b^	0.76	0.62	−0.45, 1.97	1.23	0.218
Tickling [Y]^b^	0.18	0.42	−0.65, 1.01	0.43	0.663
Sex combination [MIX]^b^	0.46	0.71	−0.94, 1.85	0.64	0.521
Sex combination [MM]^b^	0.58	0.96	−1.30, 2.46	0.60	0.545
Age‐class combination [Different]^b^	−0.51	0.74	−1.97, 0.94	−0.69	0.489
Kinship [1]^b^	0.26	0.68	−1.06, 1.59	0.39	0.695
Kinship [2]^b^	0.01	0.75	−1.45, 1.47	0.02	0.987
Object play × Tickling	−2.76	0.91	−4.55, −0.97	−3.03	**0.002**
**GLMM**_**1c**_ **—Total pause duration:** ***n*** = **107; full vs. null: *χ* ^2^** = **22.34; *df*** = **4;** ***p*** < **0.001**					
(Intercept)	0.19	0.60	a	a	a
Object play [Y]^b^	−0.69	0.38	−1.43, 0.05	−1.82	0.069
Tickling [Y]^b^	−1.21	0.27	−1.75, −0.68	−4.45	**< 0.001**
Sex combination [MIX]^b^	−0.01	0.47	−0.93, 0.92	−0.01	0.989
Sex combination [MM]^b^	0.70	0.56	−0.40, 1.80	1.25	0.213
Age‐class combination [Different]^b^	−0.55	0.41	−1.36, 0.26	−1.34	0.181
Kinship [1]^b^	0.03	0.43	−0.80, 0.87	0.08	0.938
Kinship [2]^b^	−0.13	0.47	−1.05, 0.80	−0.27	0.786
Object play × Tickling	0.26	0.55	−0.80, 1.33	0.48	0.629
**GLMM**_**1d**_ **—Total pause number:** ***n*** = **215; full vs. null: *χ* ^2^** = **8.88; *df*** = **4;** ***p*** = **0.064**					
**GLMM**_**2a**_ **—Play Asymmetry Index:** ***n*** = **45; full vs. null: *χ* ^2^** = **6.99; *df*** = **1;** ***p*** = **0.008**					
(Intercept)	−4.02	0.27	a	a	a
Tickling [Y]^b^	−0.98	0.37	−1.70, −0.26	−2.68	**0.007**
**GLMM**_**2b**_ **—Shannon Diversity Index:** ***n*** = **45; full vs. null: *χ* ^2^** = **3.85; *df*** = **1;** ***p*** = **0.0496**					
(Intercept)	−2.36	0.20	a	a	a
Tickling [Y]^b^	−0.53	0.27	−1.06, −0.01	−2.01	**0.045**
**GLMM**_**2c**_ **—Repetition Index:** ***n*** = **45; full vs. null: *χ* ^2^** = **6.58; *df*** = **1;** ***p*** = **0.010**					
(Intercept)	−4.00	0.22	a	a	a
Tickling [Y]^b^	−0.79	0.30	−1.38, −0.21	−2.66	**0.008**
**GLMM**_**2d**_ **—Pielou Evenness Index:** ***n*** = **45; full vs. null: *χ* ^2^** = **6.67; *df*** = **1;** ***p*** = **0.010**					
(Intercept)	−2.13	0.25	a	a	a
Tickling [Y]^b^	−0.77	0.29	−1.34, −0.19	−2.63	**0.009**
**GLMM**_**2e**_ **—Play Face Reciprocity Index:** ***n*** = **45; full vs null: *χ* ^2^** = **3.71; df** = **1;** ***p*** = **0.054**					
**GLMM**_**2f**_ **—Rapid Facial Mimicry:** ***n*** = **45; full vs null: *χ* ^2^** = **0.24; df** = **1;** ***p*** = **0.626**					
**GLMM**_**2g**_ **‐ Mutual gaze:** ***n*** = **45; full vs null: *χ* ^2^** = **4.70; *df*** = **1;** ***p*** = **0.030**					
(Intercept)	−1.46	0.65	a	a	a
Tickling [Y]^b^	−1.58	0.79	−3.12, −0.04	−2.01	**0.045**

*Note:* Bold values indicate *p* < 0.05.

^a^ Intercepts are shown for completeness but have no direct biological interpretation.

^b^ Categorical predictors were dummy‐coded, with reference categories set as follows: object play = absence (N), tickling = absence (N), sex combination = FF, age‐class combination = same, and kinship = non‐kin (0). All parameter estimates represent contrasts relative to these reference categories.

## 3 Results

### 3.1 Effect of Tickling on Play Sessions

GLMM_1a_ (*n* = 215) tested the effects of object play, tickling, their interaction, sex combination, age difference, and kinship on play session duration. The full model significantly improved over the null model (likelihood ratio test: *χ* ^2^ = 30.15; *df* = 4, *p* < 0.001; Table 4). Tickling was a significant positive predictor of play duration (*p* < 0.001, Table 4, Figure 2), indicating longer play sessions when tickling was present. All other predictors were not significant.

**Figure 2:**
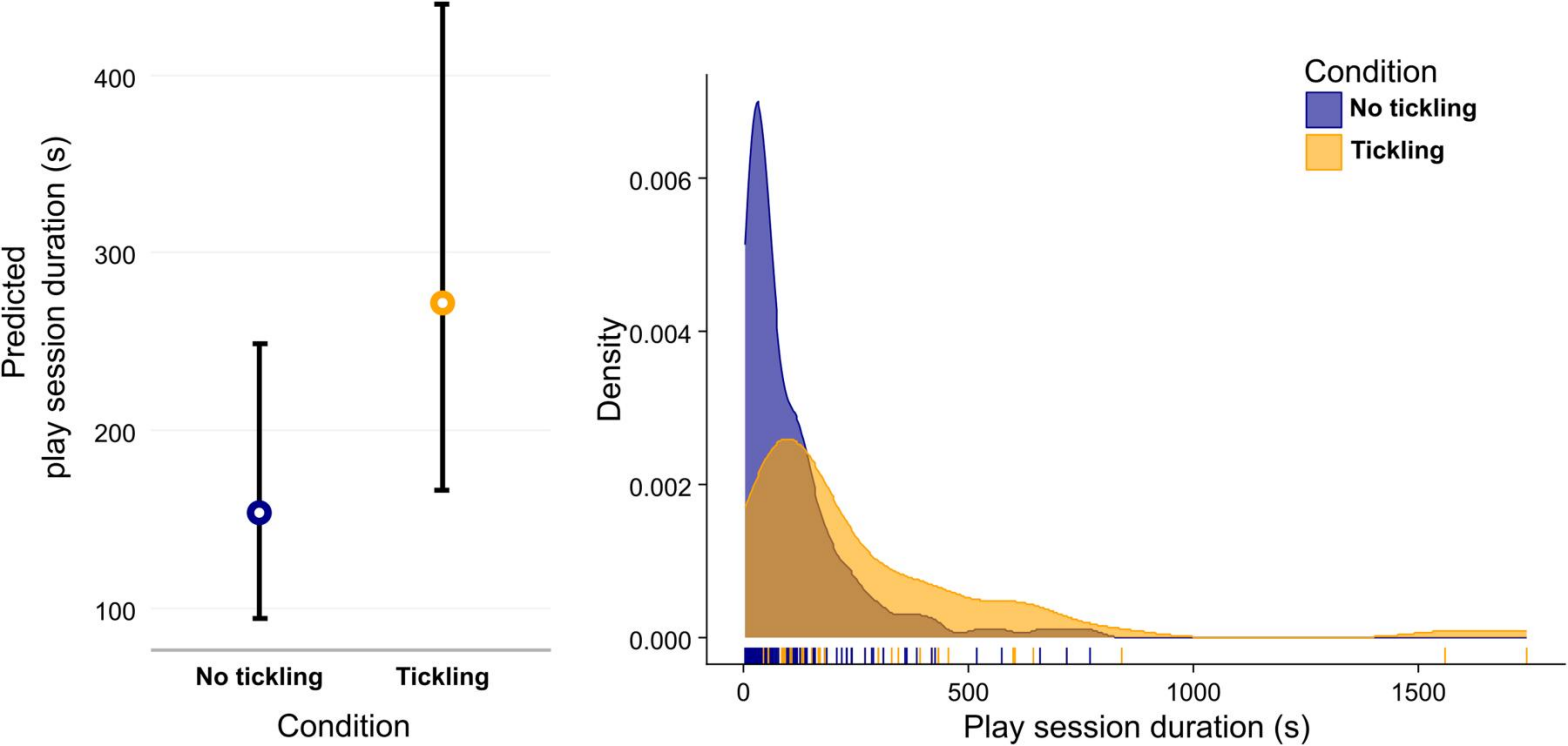
On the left, point‐range effects plot showing the estimated play session duration according to tickling condition. The *y*‐axis represents the model‐predicted play duration in seconds, points represent the model‐predicted values, and the error bars indicate the 95% confidence intervals. On the right, density plot showing the probability distribution of the observed play‐session duration. The *y*‐axis represents the probability density, and individual observations are marked by the rug plot along the *x*‐axis.

GLMM_1b_ (*n* = 215) tested the same set of predictors on the probability of pause occurrence, with play‐minus‐pause duration included as an offset. The full model significantly differed from the null model (likelihood ratio test: *χ* ^2^ = 10.69; *df* = 4; *p* = 0.030; Table 4). A significant interaction between object play and tickling was found (*p* = 0.002; Table 4, Figure 3), indicating that the effect of tickling on pause occurrence depended on whether object play was present. All other predictors were not significant.

**Figure 3:**
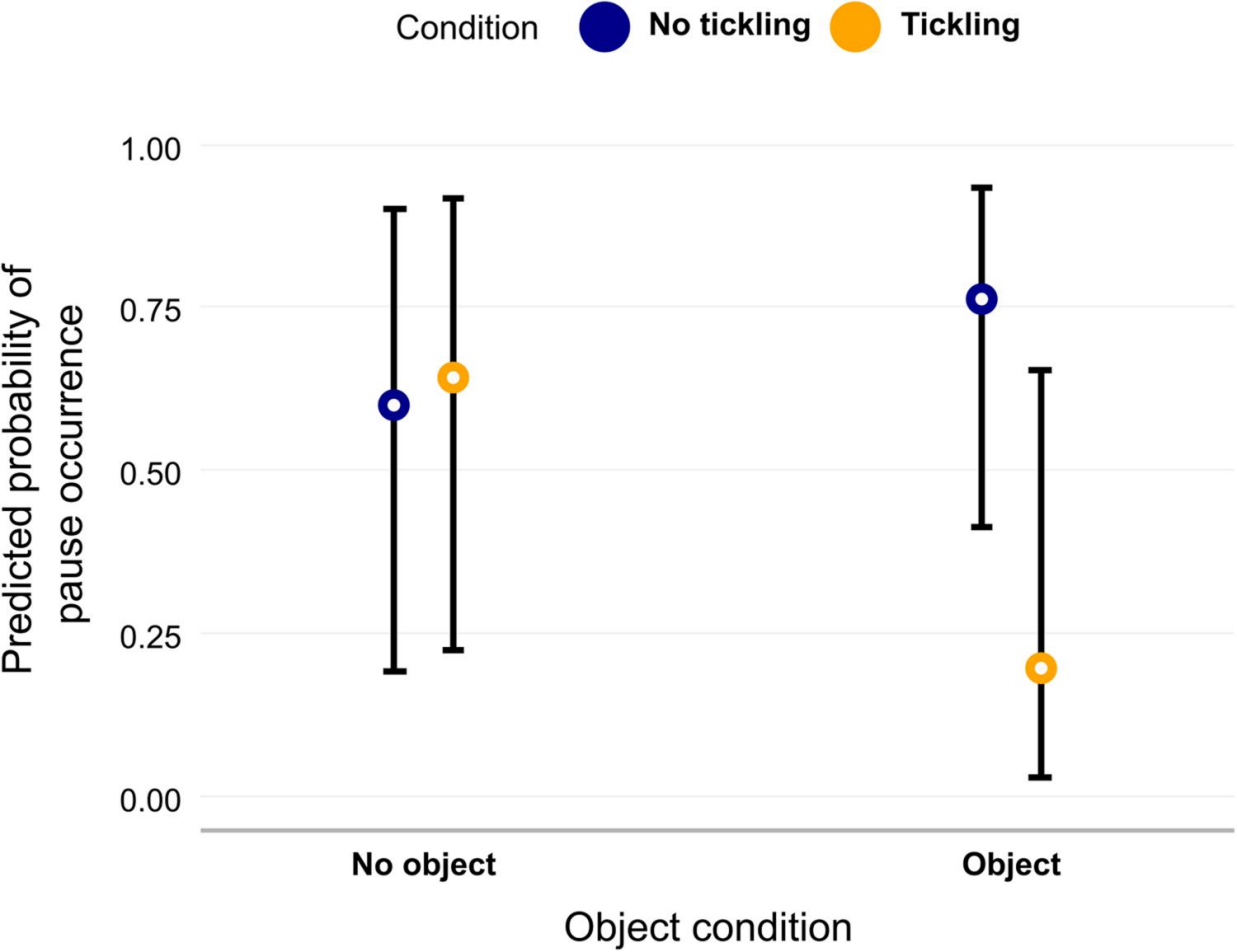
Point‐range effects plot showing the estimated probability of pause occurrence according to object condition (no object vs. object) and tickling condition (no tickling vs. tickling). The y‐axis represents the model‐predicted probability of pause occurrence, points represent the model‐predicted values, and the vertical error bars indicate the 95% confidence intervals.

GLMM_1c_ (*n* = 107) tested the effects of the same predictors on total pause duration (restricted to sessions with at least one pause), including play‐minus‐pause duration as an offset. The full model significantly improved over the null model (likelihood ratio test: *χ* ^2^ = 22.34; *df* = 4; *p* < 0.001; Table 4). Tickling was a significant negative predictor of total pause duration (*p* < 0.001; Table 4, Figure 4), indicating a shorter pause when tickling occurred. All other predictors were not significant.

**Figure 4:**
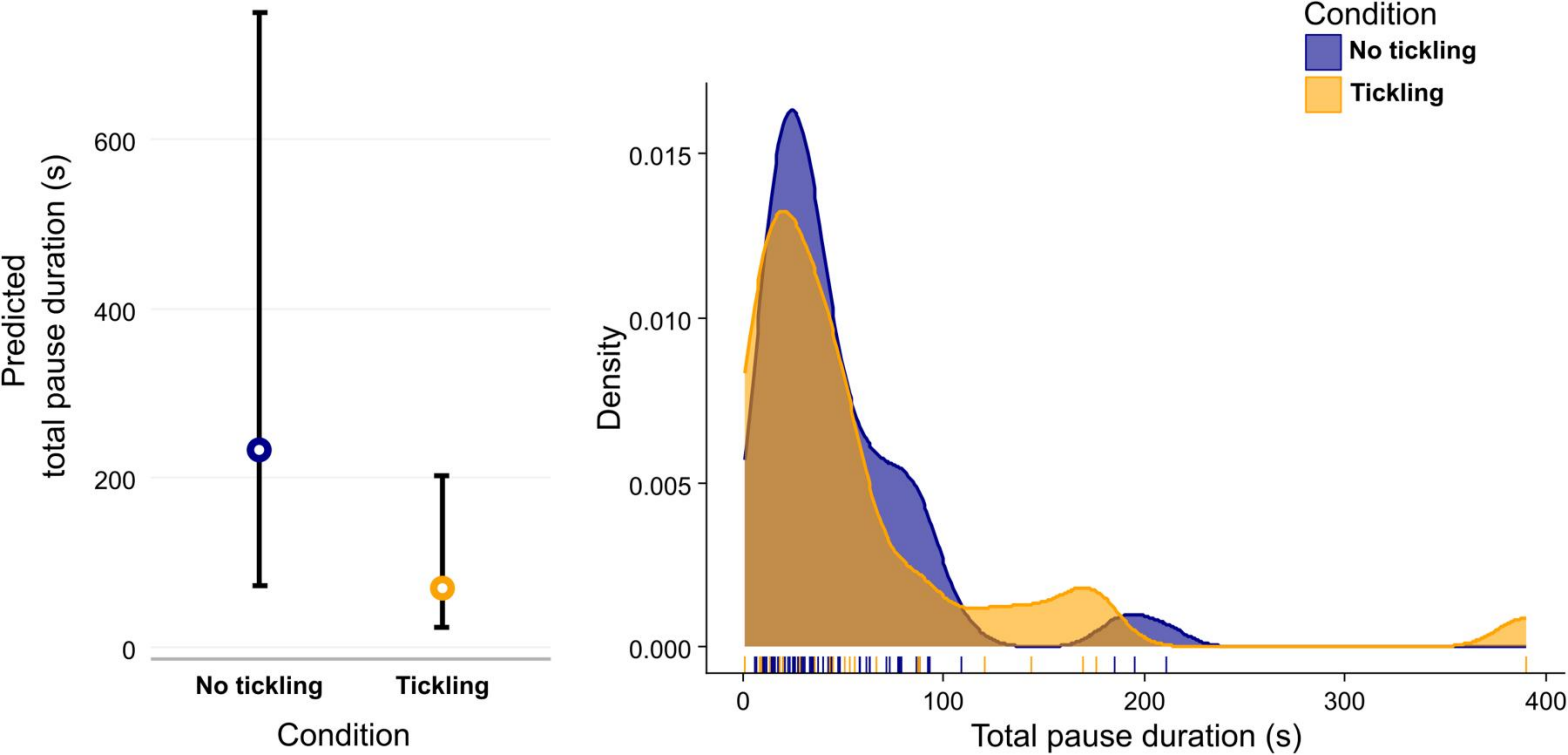
On the left, point‐range effects plot showing the estimated total pause duration according to tickling condition. The *y*‐axis represents the model‐predicted total pause duration in seconds, points represent the model‐predicted values, and the error bars indicate the 95% confidence intervals. On the right, density plot showing the probability distribution of the observed total pause duration. The *y*‐axis represents the probability density, and individual observations are marked by the rug plot along the *x*‐axis.

GLMM_1d_ (*n* = 215) tested the same predictors on the number of pauses using a negative binomial distribution. The full model did not significantly differ from the null model (likelihood ratio test: *χ* ^2^ = 8.88; *df* = 4; *p* = 0.064; Table 4), indicating that the predictors did not explain variation in pause number. Thus, none of the predictors, including object play presence, tickling, their interaction, sex combination, age‐class combination, or kinship, were significant.

### 3.2 Effect of Tickling on Play Session Structure

GLMM_2a_ (*n* = 45) tested the effect of tickling on the pAI. The full model significantly improved over the null model (likelihood ratio test: *χ* ^2^ = 6.99, *df* = 1, *p* = 0.008; Table 4). Tickling was a significant negative predictor of pAI (*p* = 0.007; Table 4, Figure 5), indicating lower pAI values in the presence of tickling.

**Figure 5:**
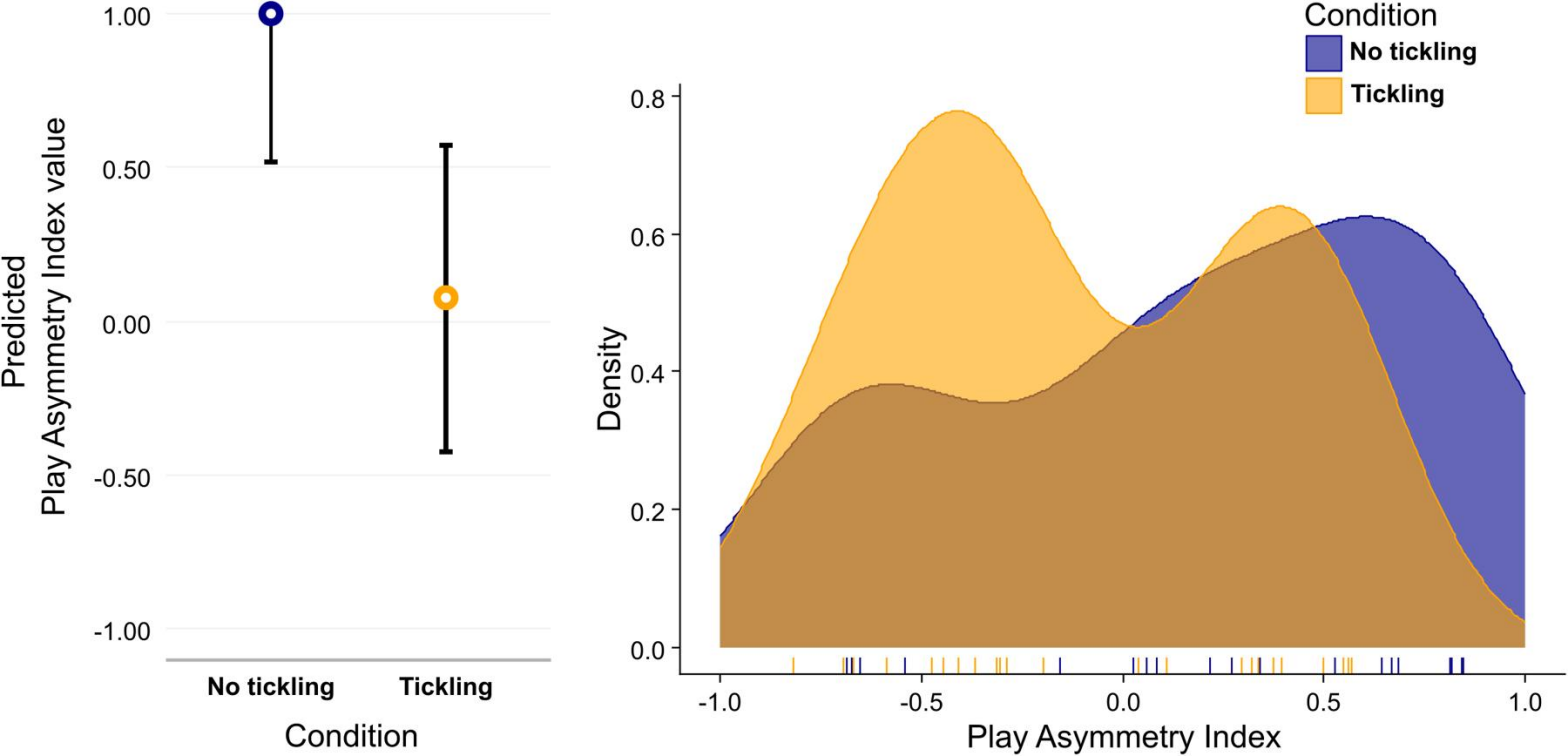
On the left, point‐range effects plot showing the estimated Play Asymmetry Index according to tickling condition. The *y*‐axis represents the model‐predicted Play Asymmetry Index value, points represent the model‐predicted values, and the error bars indicate the 95% confidence intervals. On the right, density plot showing the probability distribution of the observed Play Asymmetry Index. The *y*‐axis represents the probability density, and individual observations are marked by the rug plot along the *x*‐axis.

GLMM_2b_ (*n* = 45) tested the effect of tickling on the H′. The full model significantly differed from the null model (likelihood ratio test: *χ* ^2^ = 3.85, *df* = 1, *p* = 0.0496; Table 4). Tickling was a significant negative predictor of H′ (*p* = 0.045; Table 4, Figure 6), indicating reduced diversity during tickling sessions.

**Figure 6:**
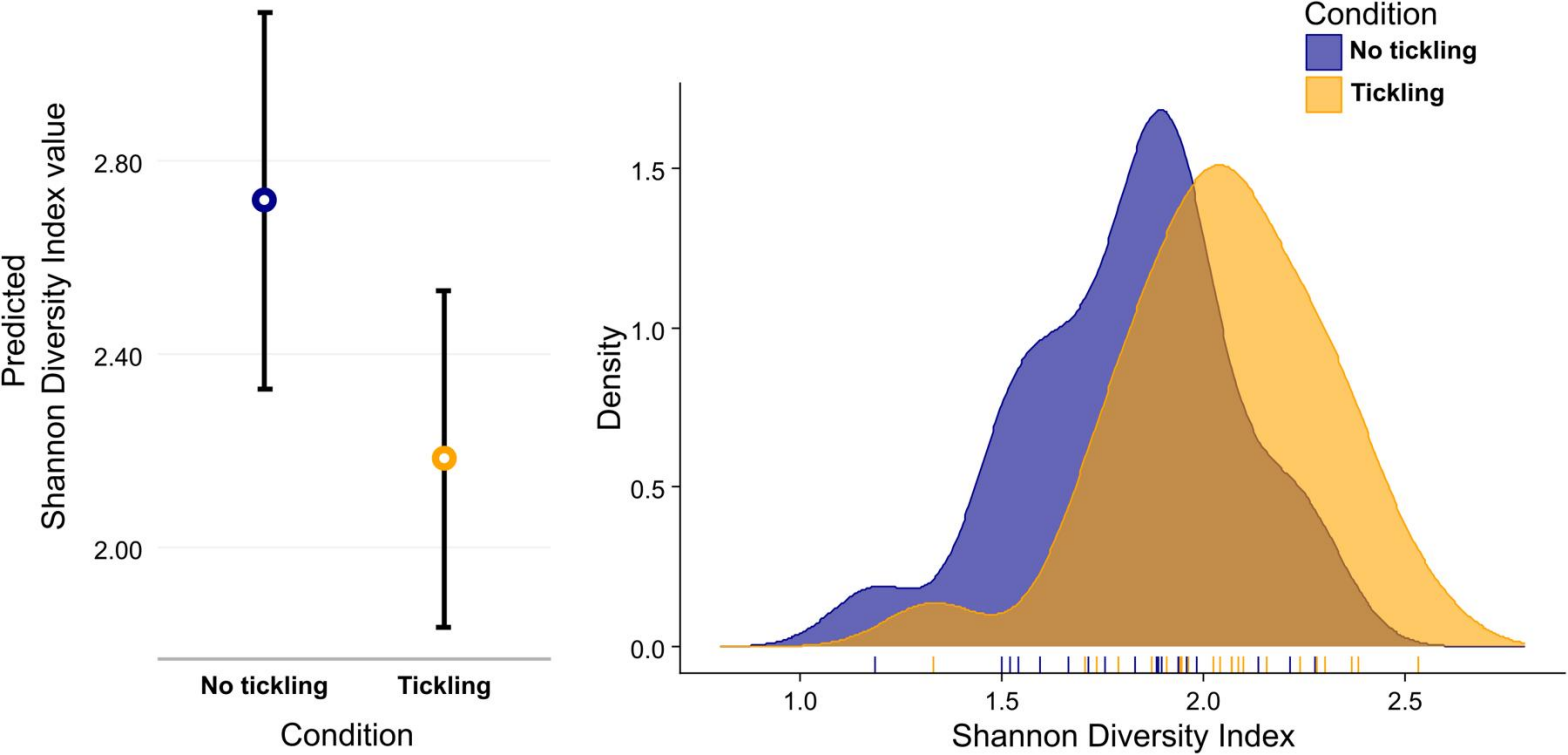
On the left, point‐range effects plot showing the estimated Shannon Diversity Index according to tickling condition. The *y*‐axis represents the model‐predicted Shannon Diversity Index value, points represent the model‐predicted values, and the error bars indicate the 95% confidence intervals. On the right, density plot showing the probability distribution of the observed Shannon Diversity Index. The *y*‐axis represents the probability density, and individual observations are marked by the rug plot along the *x*‐axis.

GLMM_2c_ (*n* = 45) tested the effect of tickling on the RI. The full model significantly improved over the null model (likelihood ratio test: *χ* ^2^ = 6.58, *df* = 1, *p* = 0.010; Table 4). Tickling was a significant negative predictor of RI (*p* = 0.008; Table 4, Figure 7), indicating reduced repetition during tickling.

**Figure 7:**
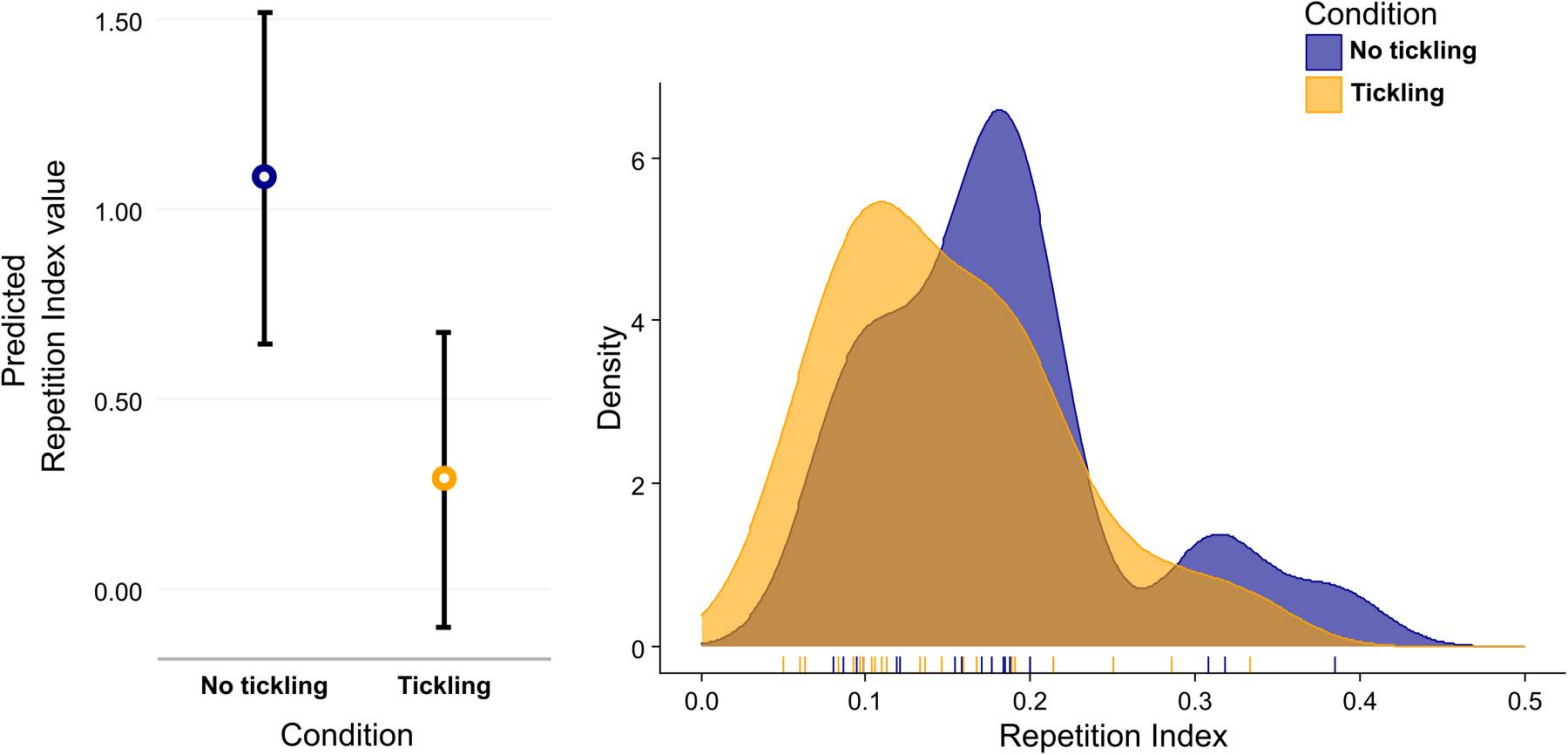
On the left, point‐range effects plot showing the estimated Repetition Diversity Index according to tickling condition. The *y*‐axis represents the model‐predicted Repetition Index value, points represent the model‐predicted values, and the error bars indicate the 95% confidence intervals. On the right, density plot showing the probability distribution of the observed Repetition Diversity Index. The *y*‐axis represents the probability density, and individual observations are marked by the rug plot along the *x*‐axis.

GLMM_2d_ (*n* = 45) tested the effect of tickling on the J. The full model significantly differed from the null model (likelihood ratio test: *χ* ^2^ = 6.67, *df* = 1, *p* = 0.010; Table 4). Tickling was a significant negative predictor of J (*p* = 0.009; Table 4, Figure 8), indicating lower evenness in tickling sessions.

**Figure 8:**
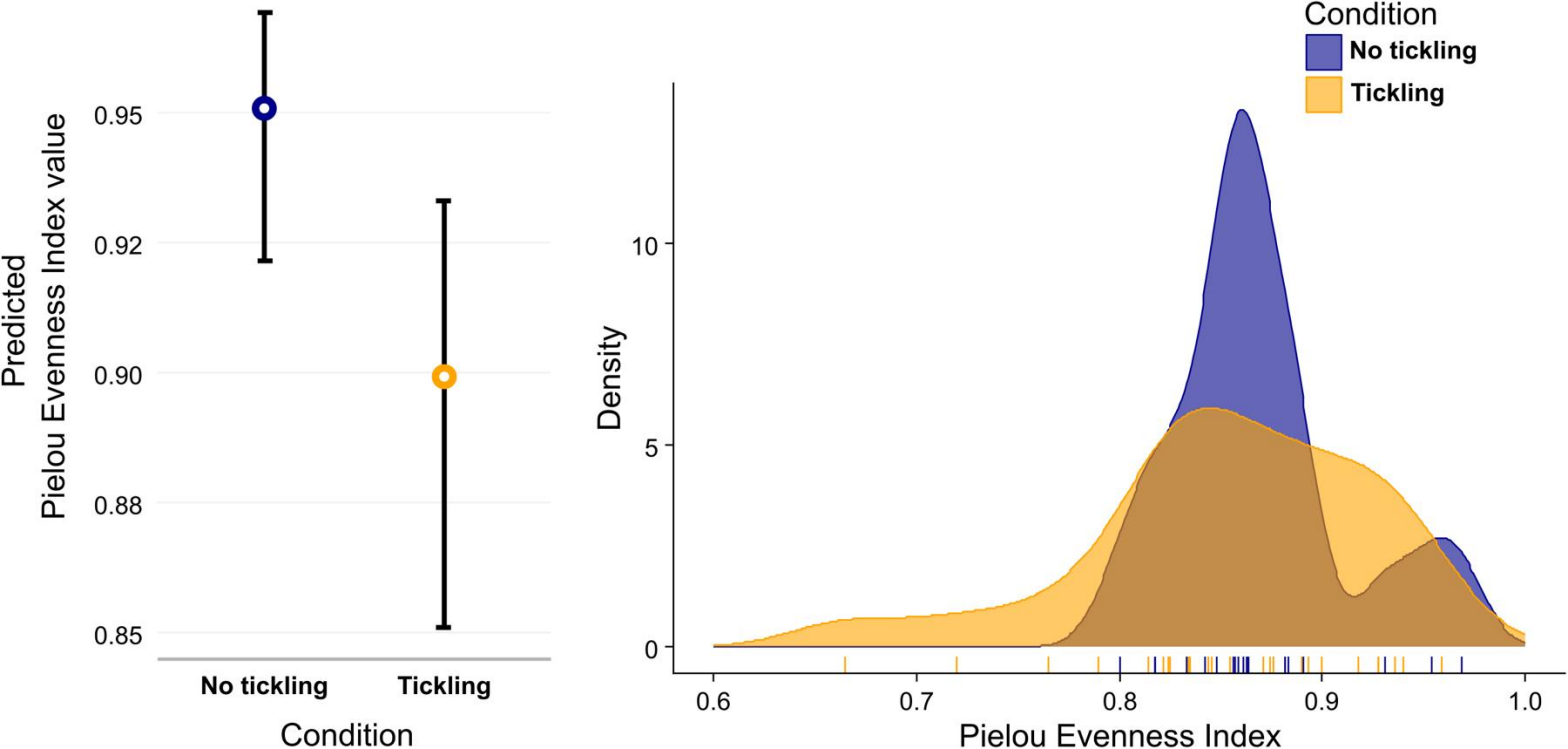
On the left, point‐range effects plot showing the estimated Pielou Evenness Index according to tickling condition. The *y*‐axis represents the model‐predicted Pielou Evenness Index value, points represent the model‐predicted values, and the error bars indicate the 95% confidence intervals. On the right, density plot showing the probability distribution of the observed Pielou Evenness Index. The *y*‐axis represents the probability density, and individual observations are marked by the rug plot along the *x*‐axis.

### 3.3 Effect of Tickling on Facial Communication

GLMM_2e_ (*n* = 45) tested the effect of tickling on the PFRI. The full model did not significantly differ from the null model (likelihood ratio test: *χ* ^2^ = 3.71, *df* = 1, *p* = 0.054; Table 4). Thus, tickling was not a significant predictor of PFRI.

GLMM_2f_ (*n* = 45) tested the effect of tickling on RFM. The full model did not significantly differ from the null model (likelihood ratio test: *χ* ^2^ = 0.24, *df* = 1, *p* = 0.626; Table 4). Thus, tickling was not a significant predictor of RFM.

GLMM_2g_ (*n* = 45) tested the effect of tickling on mutual gaze frequency. The full model significantly improved over the null model (likelihood ratio test: *χ* ^2^ = 4.70, *df *= 1, *p* = 0.030; Table 4). Tickling was a significant negative predictor of mutual gaze (*p* = 0.045; Table 4, Figure 9), indicating reduced mutual gaze during tickling sessions.

**Figure 9:**
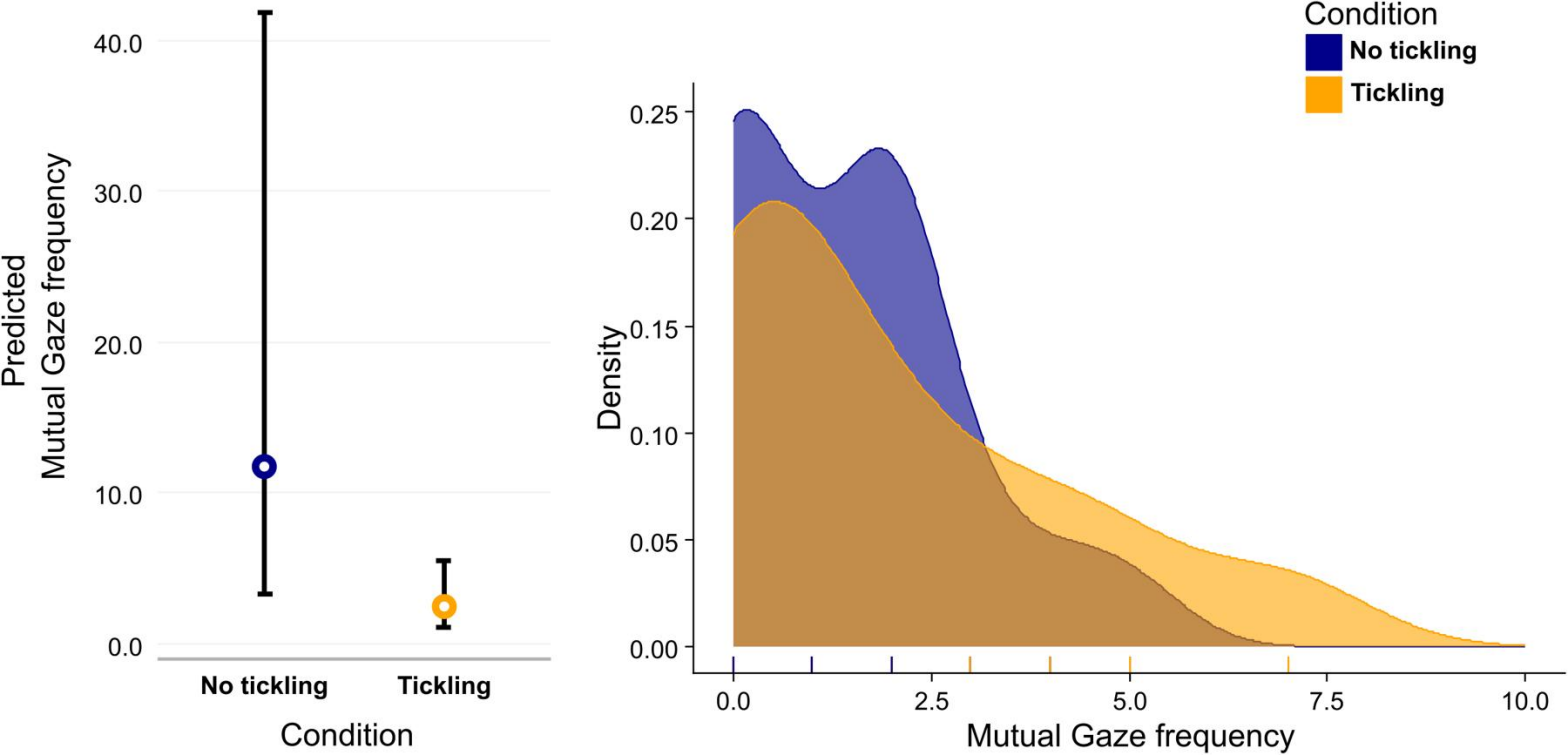
On the left, point‐range effects plot showing the estimated mutual gaze frequency according to tickling condition. The *y*‐axis represents the model‐predicted mutual gaze frequency, points represent the model‐predicted values, and the error bars indicate the 95% confidence intervals. On the right, density plot showing the probability distribution of the observed mutual gaze frequency. The *y*‐axis represents the probability density, and individual observations are marked by the rug plot along the *x*‐axis.

## 4 Discussion

The present study investigated whether tickling shapes the temporal, behavioral, and communicative organization of bonobo social play. Our findings indicate that tickling is associated with a distinctive organization of playful interactions across multiple levels.

At the temporal level, sessions containing tickling lasted longer and, among sessions with pauses, showed shorter total pause duration. The probability of pause occurrence depended on the interaction between tickling and object play, whereas the number of pauses was not explained by the predictors (*Prediction 1a* supported; *Prediction 1c* partially supported). Kinship had no effect on play duration, presence, number, and duration of pauses (*Prediction 1b* and *1d* not supported). At the behavioral level, tickling sessions showed lower play asymmetry (*Prediction 2a* supported), lower repetition (*Prediction 2b* not supported), and lower behavioral variability including diversity and evenness (*Prediction 2c* supported). At the communicative level, play‐face reciprocity (*Prediction 3a* not supported) and RFM (*Prediction 3b* not supported) did not differ between tickling and non‐tickling sessions, whereas mutual gaze was lower in sessions containing tickling (*Prediction 3c* not supported). Together, these findings indicate that tickling is associated with a form of play that is temporally sustained, behaviorally coordinated, and associated with a selective reduction in mutual gaze, rather than with a general reduction in facial communication.

Play sessions containing tickling lasted significantly longer than sessions without tickling, supporting the hypothesis that tickling contributes to maintaining social engagement over time (Bendas and Croy 2021; Heesen et al. 2021) This finding is in line with the idea that tickling facilitates sustained reciprocal engagement between partners, promoting continuous participation by both individuals and thereby prolonging playful interactions (Provine 1997; Bard et al. 2014; Ishijima and Negayama 2017; Demuru, Montello, et al. 2025). Consistently, in both humans and non‐human primates, social touch has been proposed as a primary channel through which individuals regulate one another's behavior and maintain reciprocal engagement during social interaction (Field 2010; Botero 2018; Cascio et al. 2019; Di Plinio et al. 2022). Rather than acting as a simple tactile stimulus, touch provides continuous sensorimotor feedback that supports behavioral adjustment and the ongoing coordination of actions between partners (Botero 2018; Di Plinio et al. 2022). Within this framework, the repeated tactile exchanges that characterize tickling can continuously reinforce reciprocal engagement, thereby contributing to the maintenance of play over time (Provine 1997; Ikeda and Itakura 2013; Botero 2018; Negayama et al. 2021).

In this study, kinship had no effect on the occurrence of tickling during play. This apparently differs from Demuru, Montello, et al. (2025), who found tickling to be more frequent in strongly bonded dyads. However, the comparison is not straightforward because social bonds in Demuru, Montello, et al. (2025) reflected both kinship and affiliative relationship quality. Moreover, Demuru, Montello, et al. (2025) focused only on play sessions containing tickling and its directionality, whereas we compared play sessions with and without tickling and focused on its occurrence. Together, these findings suggest that kinship per se does not influence the occurrence of tickling, whereas affiliative relationship quality may contribute to modulating it.

Our results show that tickling was linked to the temporal organization of play, as it was associated with shorter pauses. This suggests that interruptions during tickling are brief regulatory intervals that allow players to adjust their behavior before re‐entering the next stimulation–response cycle, rather than elements of disengagement. These repeated exchanges may facilitate rapid re‐engagement, thereby sustaining play over longer periods despite the occurrence of pauses. The significant interaction between tickling and object play suggests that the effects of tickling on play organization depended on the broader behavioral context. Object‐mediated play may introduce an additional triadic component to the interaction, because players must coordinate their actions with the partner while also orienting to, manipulating, or maintaining access to an external object. This interpretation is consistent with studies showing that social play with objects in great apes can involve shared attention to objects, gestural coordination, and re‐engagement after interruptions (Tanner and Byrne 2010), and with comparative work defining triadic engagement as the coordination of attention between a social partner and an object or event (Bard et al. 2021). In this context, pauses may function as brief regulatory intervals that allow players to reorient attention and adjust their actions before resuming the interaction. Importantly, this effect concerned the probability that a pause occurred, not the number of pauses, which was not significantly explained by the predictors. Thus, the temporal effects of tickling appear to be context‐dependent rather than fixed. Interruptions during spontaneous play, with or without tickling—including how they are distributed, how they affect play sessions and players, and the functions they may serve—remain largely unexplored in the literature. This study represents one of the first attempts to address these aspects, and future studies are needed to further investigate them.

Sessions containing tickling showed lower play asymmetry than sessions without tickling, indicating a more balanced exchange of offensive and defensive behaviors between partners. In play fighting, greater symmetry is generally interpreted as reflecting a more cooperative interaction (Kraus et al. 2019; Cordoni et al. 2023), because players continuously regulate their behavior through reciprocal adjustments (Aldis 1975; Pellis and Pellis 2017), role reversals, and self‐handicapping (Cordoni and Palagi 2016; Cordoni and Palagi 2016) to maintain play while reducing the risk of escalation into aggression (Bauer and Smuts 2007; Pellis et al. 2010; Pellis and Pellis 2017). Consistent with this interpretation, previous studies have shown that more symmetrical play sessions tend to last longer and are associated with greater cooperation across mammalian species (in macaques: Reinhart et al. 2010; in canids: Bekoff 2014; in *Sus scrofa*: Cordoni et al. 2022; in felines: Demuru, Collarini, et al. 2025). We acknowledge that the offensive–defensive distinction used as a standard to calculate play asymmetry (Cordoni and Palagi 2016; Cordoni et al. 2018) may be misleading, as the same pattern may constitute an attack, defence, or counterattack depending on the preceding exchange (Pellis et al. 2024). However—despite offensive/defensive distinction implying intentions that may not always be obvious to an observer in cognitively complex individuals—the pattern‐based categorisation used to calculate PAI indicates at minimum that tickling rebalances self‐directed actions and actions upon the partner. This is consistent with a more cooperative motor organization of play through reciprocal behavioral regulation. The structural indices further indicate that tickling reorganizes play structure. Lower Shannon diversity and Pielou evenness suggest that interactions became concentrated around a smaller and less evenly distributed set of behavioral patterns. This more restricted repertoire may partly reflect the highly repeated structure and the behavioral constraints imposed by tickling itself, which centres the interaction on tactile exchanges directed towards specific body regions. However, the RI also decreased, indicating that this reduced repertoire was not driven by rigidly repetitive form of play repetition. Instead, a smaller set of behaviors appears to be flexibly combined through continuous behavioral adjustments between partners. Similar patterns across structural indices have been interpreted as reflecting differences in the internal organization of play rather than simple changes in behavioral frequency (Collarini et al. 2022; Cordoni et al. 2022, 2023). Together with the reduction in play asymmetry, our findings suggest that tickling is associated with a more coordinated organization of play, characterized by balanced behavioral exchanges and a focused, yet behaviorally flexible, repertoire.

Contrary to our predictions, tickling was not associated with greater visual coordination. Play‐face reciprocity and RFM did not differ significantly between sessions with and without tickling, whereas mutual gaze was lower in sessions containing tickling. This pattern suggests that tickling does not broadly alter facial communicative exchanges, but specifically reduces face‐to‐face visual orientation between partners. One possible explanation is that this reduction reflects the physical dynamics of tickling: recipients may wriggle, withdraw, or protect sensitive body regions, naturally limiting opportunities for mutual gaze. At the same time, direct tactile contact may provide immediate information about the partner's actions and responses, allowing playful coordination without affecting other forms of facial communication (Botero 2018; Jensen et al. 2021; Aureli et al. 2022). Tactile and visual signals are complementary components of primate social communication, and their relative contribution can vary according to physical proximity, recipient attention, and interactional context (Roberts and Roberts 2015; Botero 2018; Aureli et al. 2022; Holler 2022) In this perspective, the lower mutual gaze observed in sessions points toward a context‐specific adjustment in how coordination is achieved once close tactile engagement is already established (Botero 2018; Heesen et al. 2021). Developmental studies similarly show that mutual gaze is only one possible marker of mutual engagement: in human infants, engagement can be expressed not only through face‐to‐face exchanges involving smiles and vocalizations, but also through tactile communication, especially in contexts of proximal caregiving (Bard et al. 2005; Keller et al. 2007; Bard 2009; Takada 2020). Thus, during tickling, mutual engagement may rely less on mutual visual exchange and more on tactile and sensorimotor exchanges. Consistently, studies on caregiver–infant tickling show that tactile exchanges can regulate attention, affect, and reciprocal engagement (Ikeda and Itakura 2013; Negayama et al. 2021), suggesting that similar tactile feedback may contribute to coordinating playful exchanges in bonobos. Further studies are needed to confirm whether this selective reduction in mutual gaze, in the absence of detectable changes in play‐face reciprocity and RFM, represents a consistent feature of tickling‐related play.

Taken together, our findings indicate that tickling—which was linked to longer play sessions— reorganizes play across multiple levels, structuring interactions that sustain engagement, shorten interruptions, and promote balanced behavioral participation. Tickling was also associated with a more focused, yet non‐repetitive, behavioral organization, as shown by lower diversity, evenness, and repetition. At the same time, tickling was associated with a selective reduction of mutual visual orientation between partners, rather than with a general decrease in facial communication, possibly because coordination mostly relies on tactile exchanges.

Social play has been proposed as a privileged context for investigating social cognition, communication, and the evolutionary origins of shared intentionality because it requires dyadic attunement, communicative coordination, and commitment to shared goals, including the prolongation of the playful interaction and the prevention of escalation into aggression (Bekoff 1984; Lewis 2000; Lewis and Barton 2004; Waller and Dunbar 2005; Cordoni and Palagi 2011; Heesen et al. 2021; Cordoni et al. 2022; Demuru et al. 2022). Our findings extend this perspective by informing that tickling, as a specific tactile component of social play, can offer a particularly informative context for investigating how tactile engagement contributes to the maintenance, coordination, and sensory organization of playful interactions. From this perspective, tickling may represent a valuable natural model for investigating the evolutionary foundations of joint engagement and intersubjective processes in great apes and humans, including different human cultures.

## Author Contributions

**Elisa Demuru:** conceptualization, methodology, formal analysis, writing – original draft, writing – review and editing, supervision, funding acquisition. **Chiara Andreozzi:** data collection, video‐analyses and data curation, investigation, writing – original draft, writing – review and editing. **Edoardo Collarini:** data curation, formal analysis, writing – original draft, writing – review and editing; supervision. **Marta Panunzi:** data collection, video‐analyses, investigation. **Jean‐Pascal Guéry:** logistic support at La Vallée des Singes. **Giada Cordoni:** methodology, writing – original draft, writing – review and editing. **Ivan Norscia:** conceptualization, methodology, formal analysis, writing – original draft, writing – review and editing, supervision, funding acquisition.

## Conflicts of Interest

The authors declare no conflicts of interest.

## Declaration of AI USE

We have not used AI‐assisted technologies in creating this article.

## Supporting information


**Figure S1:** Inter‐observer reliability for the recorded behavioural categories.
**Figure S2:** Empirical and fitted theoretical distributions of the target variables describing play‐session dynamics.
**Figure S3:** Empirical and fitted theoretical distributions of the target variables describing the structural indices of play sessions.
**Figure S4:** Empirical and fitted theoretical distributions of the target variables describing facial communication during play sessions.
**Table S1:** Goodness‐of‐fit statistics and Akaike Information Criterion (AIC) values for the candidate theoretical distributions of the target variables.

**Video S1:** Mother–infant tickling interactions in bonobos. The video includes two 30‐s sequences separated by a white screen, showing an adult female tickling her infant with the mouth in the first sequence and with the hands in the second. In both cases, the infants respond with play shelter and play wriggle, illustrating protective and escape‐like responses elicited during tickling stimulation.

## Data Availability

All data supporting the findings of this study are provided as Supporting Information.
