# Supplementary material for: A Ticklish Affair: Tickling Impacts Play Features in Bonobos
Source: Am J Primatol. 2026 Oct 23;88(10):e70216. doi: 10.1002/ajp.70216 (PMC13691923; doi:10.1002/ajp.70216)
Supplement: Supplementary file 1 [file AJP-88-e70216-s001.docx]

**Supporting information**

**Dataset_glmm1:** Data used for the GLMM_1_ analysis (comprising four models).

**Dataset_glmm2:** Data used for the GLMM_2_ analysis (comprising seven models).

**Video S1.** Mother–infant tickling interactions in bonobos. The video includes two 30-s sequences separated by a white screen, showing an adult female tickling her infant with the mouth in the first sequence and with the hands in the second. In both cases, the infants respond with play shelter and play wriggle, illustrating protective and escape-like responses elicited during tickling stimulation.

**Figure S1. Inter-observer reliability for the recorded behavioural categories.** The table shows the total number of observations (*N*) for each behaviour, Cohen's *K* coefficient (inter-observer agreement index), and the confusion matrix expressed as percentages of agreement and disagreement between observers.

**Figure S2.** Empirical and fitted theoretical distributions of the target variables describing play-session dynamics. **a)** Comparison of the empirical distribution of play-session duration (seconds) against Gamma, Lognormal, and Normal theoretical models, where the y-axis represents the probability density. **b)** Comparison of the empirical distribution of total pause duration (seconds) against Gamma, Lognormal, and Normal theoretical models, where the y-axis represents the probability density. **c)** Comparison of the empirical discrete distribution of the number of pauses against Negative Binomial (NegBin) and Poisson theoretical models, where the y-axis represents the probability of occurrence. The Akaike Information Criterion (AIC) values for each candidate distribution are displayed in the top-right corner of each panel.

**Figure S3.** Empirical and fitted theoretical distributions of the target variables describing the structural indices of play sessions. **a)** Comparison of the empirical distribution of the Play Asymmetry Index against Gaussian and Logistic theoretical models, where the y-axis represents the probability density. **b)** Comparison of the empirical distribution of the Shannon Diversity Index against Gamma, Lognormal, and Gaussian theoretical models, where the y-axis represents the probability density. **c)** Comparison of the empirical distribution of the Repetition Index against Beta and Gaussian theoretical models, where the y-axis represents the probability density. **d)** Comparison of the empirical distribution of the Pielou Evenness Index against Beta and Gaussian theoretical models, where the y-axis represents the probability density. The Akaike Information Criterion (AIC) values for each candidate distribution are displayed in the top-right corner of each panel.

**Figure S4.** Empirical and fitted theoretical distributions of the target variables describing facial communication during play sessions. **a)** Comparison of the empirical distribution of the Play Face Reciprocity Index against Beta and Gaussian theoretical models, where the y-axis represents the probability density. **b)** Comparison of the empirical discrete distribution of Rapid Facial Mimicry against Negative Binomial (NegBin) and Poisson theoretical models, where the y-axis represents the probability of occurrence. **c)** Comparison of the empirical discrete distribution of Mutual Gaze against Negative Binomial (NegBin) and Poisson theoretical models, where the y-axis represents the probability of occurrence. The Akaike Information Criterion (AIC) values for each candidate distribution are displayed in the top-right corner of each panel.

**Table S1.** Goodness-of-fit statistics and Akaike Information Criterion (AIC) values for the candidate theoretical distributions of the target variables. Kolmogorov-Smirnov (KS) and Anderson-Darling (AD) statistics and corresponding *p*-values are reported for continuous distributions.

| **Variable** | **Distribution** | **KS statistic** | **KS *p*-value** | **AD statistic** | **AD *p*-value** | **AIC** |
| --- | --- | --- | --- | --- | --- | --- |
| **Play session duration** | Gamma | 0.099 | 0.0294 | 2.950 | 0.0291 | 2.599.698 |
|  | **Lognormal** | **0.047** | **0.7255** | **0.384** | **0.8643** | **2.566.298** |
|  | Gaussian | 0.246 | <0.0001 | 22.011 | <0.0001 | 2.929.872 |
| **Total pause duration** | **Gamma** | **0.086** | **0.4016** | **1.223** | **0.2587** | **1.047.050** |
|  | Lognormal | 0.058 | 0.8693 | 0.246 | 0.9726 | 1.037.549 |
|  | Gaussian | 0.197 | 0.0005 | 8.590 | 0.0001 | 1.158.183 |
| **Total pause number** | Poisson | * | * | * | * | 746.573 |
|  | **Negative Binomial** | * | * | * | * | **621.143** |
| **Play Asymmetry Index** | **Gaussian** | **0.125** | **0.4431** | **1.028** | **0.3427** | **72.163** |
|  | Logistic | 0.127 | 0.4299 | 1.036 | 0.3385 | 76.835 |
| **Shannon Diversity Index** | **Gaussian** | **0.091** | **0.8173** | **0.246** | **0.9726** | **17.008** |
|  | Lognormal | 0.123 | 0.4630 | 0.568 | 0.6782 | 21.300 |
|  | Gamma | 0.113 | 0.5786 | 0.433 | 0.8152 | 19.504 |
| **Repetition index** | Beta | 0.108 | 0.6298 | 0.480 | 0.7664 | -110.569 |
|  | **Gaussian** | **0.139** | **0.3208** | **0.960** | **0.3786** | **-103.474** |
| **Pielou evenness index** | **Beta** | **0.103** | **0.6907** | **0.613** | **0.6349** | **-128.170** |
|  | Gaussian | 0.102 | 0.6957 | 0.539 | 0.7061 | -125.585 |
| **Play Face Reciprocity Index** | Beta | 0.205 | 0.0385 | 1.427 | 0.1950 | -80.164 |
|  | **Gaussian** | **0.204** | **0.0406** | **1.431** | **0.1940** | **-80.019** |
| **Rapid Facial Mimicry** | Poisson | * | * | * | * | 43.437 |
|  | **Negative Binomial** | ***** | ***** | ***** | ***** | **45.087** |
| **Mutual gaze** | Poisson | * | * | * | * | 179.787 |
|  | **Negative Binomial** | ***** | ***** | ***** | ***** | **164.493** |

Note: Asterisks (*) indicate that goodness-of-fit statistics were not computed for discrete count distributions (Poisson and Negative Binomial). The distribution family selected for the final generalized linear mixed models (GLMMs) is highlighted in bold.
